# A commentary of “Breaking the matter-antimatter mirror symmetry”： 10 remarkable discoveries from 2020 in *Nature*

**DOI:** 10.1016/j.fmre.2022.01.007

**Published:** 2022-01-29

**Authors:** Jun Cao

**Affiliations:** Institute of High Energy Physics, Chinese Academy of Sciences (CAS), Beijing 100049, China

## Abstract

The T2K collaboration reported that leptons appear to violate the “particle-antiparticle mirror symmetry”, which is also known as charge-parity (CP) symmetry [1]. Leptonic CP violation can be detected with neutrinos. Neutrinos are classified into three “flavors” based on their corresponding charged leptons (electron, muon, and tau). In-flight, they can transform from one flavor to another. If CP symmetry is conserved, the oscillation probability of transition from muon neutrino to electron neutrino will be the same as the probability of transition from muon antineutrino to electron antineutrino. In the T2K experiment, neutrinos (or antineutrinos) traveled 295 km across the earth and were then detected by an underground detector at the Kamioka Laboratory in Japan. The experiment measured the oscillation probability of transition from muon neutrino to electron neutrino as well as the transition of the antineutrinos; the results ruled out CP conservation at the 95% confidence level. This could be the first time we have found an indication of the origin of matter-antimatter asymmetry in the universe.①

In the very early stages of the birth of the universe, energy transformed into matter. According to the theory of particle physics, the number of matter and antimatter particles produced should be equal. Antimatter is almost negligible in our current universe compared with the matter. It is generally believed that something happened 10^–12^ s after the birth of the universe that caused the asymmetry between matter and antimatter of the universe. In 1967, A. Sakharov of the former Soviet Union and considered as the “father of the Soviet hydrogen bomb” proposed three conditions for the creation of asymmetry between matter and antimatter in the universe: the existence of a baryon number non-conservation process, charge symmetry and CP symmetry violation, and deviation from thermal equilibrium. These three conditions can be met in principle in the Standard Model of particle physics; however, the exact mechanism is still unknown.

CP symmetry is conserved in the vast majority of particle physics processes. CP non-conservation may occur in the presence of three generations of quarks (Nobel Prize in Physics in 2008). To measure CP non-conservation in quarks, the United States and Japan built the Babar and Belle experiments. The three mixing angles and CP phase are related to the magnitude of the CP violation effect. It has been discovered experimentally that the three mixing angles of quarks are very small. Therefore, the total CP violation effect is also very small— one million times smaller than the effect required to explain the asymmetry between matter and antimatter in the universe. The discovery of neutrino oscillation 20 years ago (for which the Nobel Prize in Physics was awarded in 2015) has given rise to new hope. Neutrino oscillations will also cause CP non-conservation. The Super-Kamiokande experiment in Japan, the Sudbury Neutrino Observatory in Canada, and the Daya Bay experiment in China, together with many other experiments, have measured respectively the three mixing angles of neutrinos, which are much larger than the mixing angles in quarks. Thus, if the CP phase of the neutrino is also large, a large asymmetry between matter and antimatter can be produced. This mechanism is known as the leptogenesis, and it appears to be the most natural explanation for the mysterious disappearance of antimatter in the universe.

The T2K experiment uses the Japanese spallation neutron source accelerator to generate a beam of muon neutrinos that was fired at the 50,000 tons Super-Kamiokande neutrino detector 295 km away. During the flight, a small part of muon neutrinos spontaneously transforms into electron neutrinos, which is known as the neutrino oscillation. By switching the electric current direction of the focusing magnet on the beam, neutrinos or antineutrino beams can be generated. If the CP violation is not zero, the probability of oscillation of neutrino and antineutrino is different such that the asymmetry between matter and antimatter caused by neutrino oscillation can be directly observed.

In the T2K experiment in 2011, that had just been put into operation, the conversion of muon neutrinos into electron neutrinos was observed for the first time, indicating that the neutrino mixing angle θ_13_ could be quite large. Unfortunately, the “March 11^th^” earthquake in Japan damaged its accelerator, making it impossible to obtain a definitive result in short term. In March of the following year, the Daya Bay experiment in China was the first to discover the 3rd neutrino oscillation mode associated with θ_13_. The data volume of T2K increased by 4.6 times from 2011 to 2014, and a definitive result that θ_13_ was not zero was also obtained. It is intriguing that T2K and Daya Bay experiments use different physical principles and methods for measuring neutrino oscillation. After combining the precise value of θ_13_ measured by the reactor neutrino experiments (mainly Daya Bay), T2K was the first to show the potential for detecting CP violation in the same paper. T2K published several CP violation measurement results after that. In 2020, T2K published the results in *Nature*, with 20 times the amount of data used in 2011, half of which is neutrino data and the rest, antineutrino data. By comparing the neutrino and antineutrino oscillations, it is found that CP is not conserved at the 95% confidence level, and the CP phase is large. This could be the first time that evidence of the origin of asymmetry between matter and antimatter in the universe has been discovered.

The T2K experiment will last until 2026, with the data volume doubling. Hopefully, the key evidence of CP non-conservation will be found (i.e., the 99.7% confidence level, a widely accepted standard in particle physics, can be reached).

The next generation of neutrino experiments, on the other hand, is required for more accurate and reliable measurements. The Jiangmen Underground Neutrino Observatory (JUNO) in China, the Deep Underground Neutrino Experiment (DUNE) in the United States, and the Hyper-K experiment in Japan are among the next-generation neutrino experiments currently under construction. JUNO will use the experiences gained from the Daya Bay experiment, primarily using reactor neutrinos. The main goal is to accurately measure the neutrino mass ordering and three oscillation parameters, but it cannot measure CP violation. The accelerator neutrino experiments DUNE and Hyper-K can compare directly the difference between the neutrino and antineutrino oscillations, thus measuring the CP violation. Very likely, we should be able to determine the size of the neutrino CP violation in the next 20 years, putting us one step closer toward understanding the origin of the universe.

## Declaration of Competing Interest

The author is a member of the Daya Bay and JUNO collaborations. The author declares that he does not have any other conflicts of interest in this work.
